# The Development of a Mobile Monitoring and Feedback Tool to Stimulate Physical Activity of People With a Chronic Disease in Primary Care: A User-Centered Design

**DOI:** 10.2196/mhealth.2526

**Published:** 2013-07-02

**Authors:** Sanne van der Weegen, Renée Verwey, Marieke Spreeuwenberg, Huibert Tange, Trudy van der Weijden, Luc de Witte

**Affiliations:** ^1^CAPHRI School for Public Health and Primary CareDepartment of Health Services ResearchMaastricht UniversityMaastrichtNetherlands; ^2^Research Centre Technology in CareZuyd University of Applied SciencesHeerlenNetherlands; ^3^CAPHRI School for Public Health and Primary CareDepartment of General PracticeMaastricht UniversityMaastrichtNetherlands

**Keywords:** user-centered design, self-management, physical activity, accelerometry, remote sensing technology, primary health care

## Abstract

**Background:**

Physical activity is an important aspect in the treatment of patients with chronic obstructive pulmonary disease or type-2 diabetes. A monitoring and feedback tool combined with guidance by a primary care provider might be a successful method to enhance the level of physical activity in these patients. As a prerequisite for useful technology, it is important to involve the end-users in the design process from an early stage.

**Objective:**

The aim of this study was to investigate the user requirements for a tool to stimulate physical activity, embedded in primary care practice. The leading principle of this tool is to change behavior by self-monitoring, goal-setting, and feedback.

**Methods:**

The research team collected qualitative data among 15 patients, 16 care professionals, and several experts. A prototype was developed in three stages. In stage 1, the literature was searched to identify end-users and context. In stage 2, the literature, experts and patient representatives were consulted to set up a use case with the general idea of the innovation. In stage 3, individual interviews and focus groups were held to identify the end-user requirements. Based on these requirements a prototype was built by the engineering team.

**Results:**

The development process has led to a tool that generally meets the requirements of the end-users. A tri-axial activity sensor, worn on the hip, is connected by Bluetooth to a smartphone. In an app, quantitative feedback is given about the amount of activity and goals reached by means of graphical visualization, and an image shows a sun when the goal is reached. Overviews about activity per half an hour, per day, week, and month are provided. In the menu of the app and on a secured website, patients can enter information in individual sessions or read feedback messages generated by the system. The practice nurse can see the results of all patients on a secure webpage and can then discuss the results and set personalized goals in consultation with the patient.

**Conclusions:**

This study demonstrates that a user-centered approach brings in valuable details (such as the requirements for feedback in activity minutes per day) to improve the fit between the user, technology, and the organization of care, which is important for the usability and acceptability of the tool. The tool embedded in primary care will be evaluated in a randomized controlled trial.

## Introduction

Lack of physical activity is an important risk factor for cardiovascular disease, hypertension, diabetes mellitus, obesity, stroke, some cancers, and osteoporosis. It is recommended that the general population is physically active at a moderate to vigorous intensity for at least 150 minutes per week [1]. Unfortunately, physical inactivity remains highly prevalent [2,3]. It is particularly important for people with a chronic disease to be physically active. It has not only been proven that an active lifestyle prevents diseases but also an active lifestyle improves the health-related quality of life and psychological status for people with a chronic disease [4,5]. An active lifestyle reduces dyspnea in chronic obstructive pulmonary disease (COPD) patients [6] and complications in patients with diabetes [7]. Due to the health benefits and the need for support by most COPD and type-2 diabetes (DM2) to increase their physical activity, stimulating physical activity is regarded as one of the main treatment goals in primary care [8,9]. This should be accomplished with support for self-management. Self-management implies that people are in charge of their own lives with their disease and its treatment, enabling motivation to change. Supporting self-management requires a different role of health care professionals and patients, for which new skills and tools are needed [10].

Professionals can be more successful at improving an active lifestyle by increasing patients’ awareness through self-monitoring, goal setting, and discussing self-efficacy [11,12]. The provision of tailored feedback on physical activity has been proven to be effective in several interventions [13-15]. Persuasive technology can help professionals in accomplishing their coaching role. A “simple” pedometer gives feedback about the frequency of steps or distance walked in a day and it seems to be a useful tool that incorporates elements for self-monitoring, goal-setting, and feedback. Self-reporting studies revealed that the use of pedometers is an effective approach to increase physical activity [16-18]. It is, however, still unknown to what extent the observed changes are sustainable or whether it is possible to continue to accumulate benefits as a result of long-term adherence [16,18]. Due to new technological developments, such as pedometers being improved to (tri-axial) accelerometers and mobile phones being transformed into mini-computers, new possibilities for activity monitoring have become available. Numerous activity monitors are commercially available [19]. For example, the Fitbit provides feedback on steps, distance, and calories [20]. The activity monitor, PAM, engages the participant by giving points for the activities in a “PAM-score” and detailing a historical overview on a personal website [21].

Furthermore, systems are developed in which pedometers/accelerometers are connected wirelessly to a mobile phone [22-24]. This connectedness with mobility makes it possible to give more detailed readily available feedback on a larger screen. Linking self-monitoring technologies with a coach or embedding such technologies in the care process could further enhance effectiveness of behavior change strategies [25-27]. especially when technology and care are carefully developed and aligned with each other.

In the project “It’s LiFe!”, an innovative monitoring and feedback tool was developed which is embedded in primary care practice. The tool aims to support the self-management of people with COPD or type-2 diabetes to obtain an active lifestyle by measuring their activity behavior, giving automatically generated tailored feedback to the patient and to the care professional. The care intervention in which the tool will be embedded is named the Self-management Support Program (SSP). The program consists of a limited number of behavior change consultations with a health care professional.

As a prerequisite for useful technology and a successful intervention that meets the requirements and preferences of end-users, it is important to involve the end-users in the design process at an early stage [28]. In the project “It’s LiFe!”, this inclusiveness of end-users was ensured by a user-centered design process in which people with COPD or type-2 diabetes and their health care professionals were involved in the development of the technology and the SSP.

The aim of this paper is to report on the user-centered design process in which the user requirements for a monitoring and feedback tool were investigated. In particular, users were involved to reveal:

1. Which feedback patients and professionals need to optimally support self-management of physical activity?
2. How this feedback can best be presented?
3. How the tool can be made attractive, persuasive, easy to use and suitable to wear on a daily basis?

## Methods

### User-Centered Design Process

A user-centered design (UCD) process was followed. User-centered design is a broad term that describes design processes in which end-users influence how a design takes shape. To increase the success rate of the usability in computerized systems [29], it is of importance to understand the context of use and the user requirements [30,31].

To ensure UCD from the outset, two patient representatives were recruited from the national patient associations for COPD and diabetes, participated in the research team. These representatives reflected on the needs, demands, and restrictions of the patients. Furthermore, the representatives provided feedback on the comprehensibility of interview questions, the use cases, and other documents which were intended for patient participants in the study. The research team gathered the user requirements and an engineering team translated these into technical solutions. During the development process for the monitoring and feedback tool, there was a continuous interaction between the research team and the engineering team. This interaction facilitated the match between the user requirements and the technical solutions.

**Figure 1:**
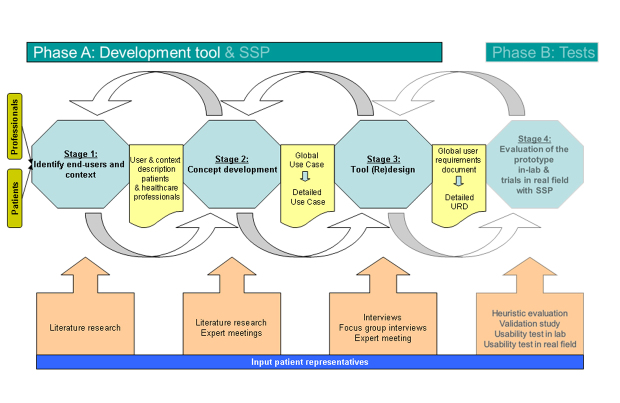
The It’s LiFe! user-centered design process. First the end-users and context were defined based on the literature. Second the conceptual idea of the tool was described in a use case, based on input from literature, an experts meeting and patient representatives. In stage 3 the use case was discussed with patients and health care professionals to elicit the user requirements for the tool. During the whole process the research team deliberated with the engineering team, to find out what was technically possible. After a detailed user requirements document was composed the engineering team translated the user requirements in technical solutions.

The design process of the monitoring and feedback tool was based on a combination of existing methodologies, but mainly on Shah’s methodological framework for medical device development [28,32-34]. The tool was developed in three iterative stages, depicted as phase A in Figure 1. The end-users, people with COPD or type-2 diabetes and their primary care professionals, were extensively involved. In the fourth stage (phase B), the tool will be tested in laboratory situations and real-life settings. The three development stages are described below.

#### Stage 1: Identify End-Users and Context

To outline the context in which the monitoring and feedback tool should be used, end-users’ and environmental characteristics were identified by analyzing the literature and clinical practice guidelines [8,9]. This resulted in a narrative description of users and context.

#### Stage 2: Concept Development

Literature was studied about behavior change strategies and technologies to stimulate physical activity that would match with users and context. The user and context description and the literature findings were discussed with experts (physicians, human-movement scientists, technicians, and implementation experts). The results of stage 2 were specified in a global use case; describing the interaction between a user and the system to be developed in a step-by-step manner [35]. The use case was designed to demonstrate the conceptual idea of the tool to end-users, without giving too much direction to their thoughts.

#### Stage 3: Tool (Re) Design

In order to elicit user requirements for the tool, the use case was the object of discussion with patients in 15 semi-structured interviews (in 2 rounds), and 2 focus group discussions were held (1 for COPD and 1 for diabetes. In the focus groups (FG), the patients discussed and complemented the interview results. After another 16 interviews with health care professionals, all of the results were discussed with the same experts from stage 2 plus an independent eHealth researcher and an opinion leader from a general practice. For the interview topics see Table 1. After the interviews and focus group discussions, a first draft of the user requirements document was established. The requirements document was deliberated upon with all of the project members, particularly with the engineering team, to confirm the technical possibilities and to ensure that no important issues were neglected.

**Table 1:** Interview topics regarding the tool for patients and professionals.

Main topics		Subtopics
**Tool architecture**		
		Place activity sensor
		Requirements activity sensor
**Goal setting**		
		What kind of goal
		Who should set the goal
		On what condition should the goal be adaptable
**Feedback**		
	1. Amount of activity	In what unit should it be presented
		Where should it be visible
		In what format should it be visible
	2. Amount of activity compared to goal	In what unit should it be presented
		Where should it be visible
		In what format should it be visible
	3. Response of a health professional based on the activity results	Which health care professional should be involved
		How should the health care professional react on the results
		How do patients feel about the possibility for a health care professional to look at their activity results
**Data sharing (only discussed with patients)**		
		Share activity results with peers
		Share activity results with relatives

### Recruitment and Data Sampling

Patients and health care professionals were recruited by snowball sampling, by using contacts from the national patient associations and the researchers’ networks. Interviews lasted approximately 90 minutes and were held in the respondents’ natural environment (at home) or at Maastricht University. Interviewees were asked to read the use case and give their opinion about the conceptual idea of the technology, integration into primary care and their specific requirements for such a tool. During the interviews, the questions and the use case were continuously specified.

### Data Analysis

All of the interviews and focus group discussions were audio-taped with the consent of the respondents. The first 8 interviews with patients and all interviews with health care professionals were transcribed verbatim. Transcripts were generated, read, and open-coded using the NVivo 2.0 software package. Two researchers independently open-coded 4 interviews (2 from patients and 2 from health care professionals) and reached a consensus about final coding with themes and subthemes. Next, all of the transcripts were (re-)coded using the themes and subthemes as an analytical coding scheme that further evolved during the analysis. After analysis of interviews for round 1 (IR1), questions for each end-user group were rephrased from open to closed questions. For example, in the first interview round the patients were asked, “where do you want to wear the activity sensor”, whereas in the interviews for round 2 (IR2) all of the previously mentioned answers which were technically possible were given for the patient to choose from. However, other additional options were also welcomed. The audio recordings of the second round of patient interviews and the focus groups were transcribed per code and analyzed by two researchers independently. By means of member check, the results of the focus group discussions were verified by a focus group participant and an observer who was present at both of the focus group discussions.

### Ethical Approval

This study was approved by the medical ethical committee of azM/UM.

## Results

### Stage 1: User and Context Description

Concerning the users and context, it was considered that the resulting intervention (tool + SSP) would be focused on anybody who will benefit from support during physical activity. However, for the scope of the research project, people with COPD or type-2 diabetes, aged less than 70 and over 40, treated in a primary care setting were chosen. These two patient groups were chosen since they represent a large part of the chronically ill people in primary care and can both benefit from lifestyle changes. More importantly, these two groups have diverse physiological and psychological states and therefore different support needs [36]. Involving this heterogeneous group in the development should lead to a tool that is applicable for a wide audience. However, this implies that the tool should have customizable components to meet the specific needs of different target groups. It was also decided to develop an intervention for people in the contemplation (thinking about change) and preparation (making small changes in behavior but not enough) phase of the Trans-theoretical Model of Behavior Change [37,38]. We believe that people in these stages benefit most from support in self-management. People in the precontemplation phase need to be convinced of the importance of an active lifestyle first. Based on the clinical practice guidelines [8,9], the practice nurse was the logical health care professional to be involved.

### Stage 2: Concept Development

#### Literature Findings

In order to develop an effective intervention, it must be clear which determinants are relevant for the target behavior and which of them can be influenced. For the initiation and maintenance of physical activity, the relevant and changeable personal determinants are: awareness, knowledge, attitude, self-efficacy, intention and intrinsic motivation [39,40]. Strategies to influence these determinants include: self-monitoring, providing tailored feedback, providing information, action planning, working with role models, and proposing activities that are feasible for the patient [40]. It is important to note that intention to change is not sufficient; intentions account for only 20-30% of the variance in behavior [41]. In order to narrow the gap between intentions and actual behavior, it is important to set realistic goals and to identify potential barriers. The Goal-Setting Theory states that a goal should be specific, challenging but realistic, set by the patient himself (or in collaboration with the health care professional), and easily monitored [42].

Physical activity can be measured with questionnaires, energy expenditure measurements and activity monitors. For daily use, activity monitors are most suitable [43]. There are three classes of activity monitors; pedometers, accelerometers and integrated multisensory systems. Pedometers estimate the number of steps taken but are limited to measurement of the vertical plane. Accelerometers detect acceleration in one, two or three directions and can determine the amount, intensity and duration of movements. Integrated multisensory systems try to optimize physical activity assessment using the combination of accelerometry and other sensors that measure physiological responses to exercise, such as skin temperature or heart rate. However, there is little evidence that adding another physiological measure significantly improves the assessment of energy expenditure [44]. Numerous accelerometers are developed with different wearing positions, such as the hip, waist, ankle, upper leg, and wrist. An accelerometer is most accurate in assessing daily life physical activity if worn on the lower back or hip [44]. However at this position, cycling is not captured very well. A promising development is monitors that integrate Global Positioning Systems [43] which could make it possible to measure cycling. Unfortunately at the moment, this is too energy consuming for daily use.

From other monitoring and feedback tools it was learnt that feedback and incentives should be provided whenever progress is made and not only when the goal is achieved [18]. From the development and evaluation of two mobile systems, Houston [23] and Ubifit [24], it can be seen that mobile interventions can be a powerful way of promoting health behavior changes. This is achieved by supporting the persistent activation of health goals, focusing on patterns of activity, and facilitating optional social support [45].

#### Global Use Case

The literature findings and meetings with experts led to the following concept of the tool. This concept of the tool was elaborated in the global use case, which was presented as a narrative scenario, and was the input for stage 3. Basically, the tool, consisting of a sensor and a feedback system, will focus on the stimulation of daily activity and not on sports. The sensor is placed somewhere on the body and measures physical activity. The patient sets a personal activity goal together with the health care professional, and receives feedback about the current activity level related to the pre-set activity goal. The health care professional and a relative also receive a periodic summary of the activities. When the patient is performing well, the patient receives compliments from the tool, the health care professional, and their relative.

### Stage 3: Tool (Re-)Design

**Figure 2:**
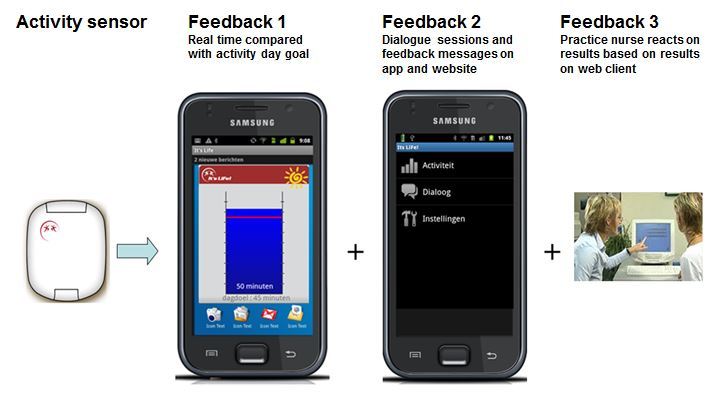
The monitoring and feedback tool that was developed, based on the requirements of the end-users. The tri-axial activity sensor is connected via Bluetooth to the smartphone. The smartphone gives directly visible feedback about the amount of activity in a bar chart, which dynamically fills up. When the goal (indicated by the red line) has been reached, a sun rises. In the app and on a secure webpage, people can see their activity history and answer dialogue sessions and read feedback messages generated by the system. The practice nurse can monitor the results of all patients on the secure web page to discuss during patient visits.

The purpose of this stage was to further specify the conceptual idea to the user requirements and preferences of the patients and health care professionals. The characteristics of patients and professionals who participated in the interviews and focus groups are described in Tables 2-4. Four main topics relevant for the tool were identified from the interviews: (1) Tool architecture; (2) Goal setting; (3) Feedback; and (4) Data sharing. For each topic, user requirements and preferences were elicited. For the resulting design of the tool and a visualization of the feedback loops (described under “feedback consequences”) see Figure 2.

**Table 2:** Characteristics of respondents with COPD from the interviews and focus groups.

	Interview round 1 (n=4)	Interview round 2 (n=3)	Focus group (n=6)
Age mean (SD)	64 (7.2)	61.5 (5.3)	61.8 (5.7)
GOLD	2-4	3-4	2-4

**Table 3:** Characteristics of respondents with DM2 from the interviews and focus groups.

	Interview round 1 (n=4)	Interview round 2 (n=4)	Focus group (n=5)
Age mean (SD)	61.5 (5.3)	62.8 (12.8)	56.8 (8.2)

**Table 4:** Characteristics of health professional respondents from the interviews and focus groups.

	Interview round 1 (n=11)	Interview round 2 (n=5)
Practice nurse	2	5
Diabetes nurse	2	0
Pulmonary nurse	2	0
General practitioner	3	0
Physiotherapist	2	0
Age mean (SD)	42 (11.5)	42 (11.8)

#### Tool Architecture

During the interviews, several requirements relevant for the activity sensor arose. The most important requirements were that it should measure all activities. The specific design was not important as long as it did not hinder movements or was obtrusive. For the location of the sensor, the hip and wrist were the most popular.

A place where it is not visible and does not hinder you. If you wear a wristwatch for example, people may ask you what it is. That is nice for the first few times, but after 13 times it is not. So something like a watch, but then for around your ankle. Or something around the belt that is always hidden.

IR2, DM2, man, 60 years

Only two respondents did not prefer a sensor on the hip, with the argument that it may be problematic for women wearing a dress and people doing many arm activities.

I think this is a man’s idea, since a woman cannot wear this sensor when they wear a dress. And people with COPD GOLD 1 or 2 do not feel sick yet, so they will not wear an inelegant device.

FG, COPD, female, 57 years

Immediate feedback should be visible at a glance. Respondents preferred to receive feedback on a mobile phone, since it has a larger screen than an activity sensor and is more readily accessible than a computer. For more comprehensive use, however, such as manual data entry and consulting activity histories, a computer was considered more feasible.

#### Consequences for Tool Architecture

Based on the results above, it was decided that the “It’s LiFe!” tool would consist of three elements (presented in Figure 2):

1. An activity sensor with Bluetooth connectivity worn on the hip and clipped on the belt.
2. A smartphone with an app for mobile feedback.
3. A Web client for comprehensive feedback and data entry for patient and practice nurse.

The “It’s LiFe!” activity sensor is a 3D accelerometer with a sample frequency of 25 Hz. This newly developed activity sensor (4.0 x 4.5 centimetre) is based on the Ciro Activity Monitor (CAM) also manufactured by Maastricht Instruments [46]. The validity of the sensor will be tested in a subsequent study on a treadmill and in free-living conditions. The smartphone and web client would be connected to a web server with facilities for data storage and feedback generation. Nearly all of the user requirements were met. For budget reasons, however, the activity sensor could not be made suitable for cycling and swimming. It was decided that the amount of swimming and cycling activities should be entered by the users manually. The final architecture of the activity sensor was chosen from two prototypes by the patient representatives.

#### Goal Setting

Patient respondents preferred to set their goals together with the practice nurse. This would prevent the under- or overestimation of their abilities. In general, daily goals were preferred since weekly goals lead to postponing activities. Some respondents with COPD, however, indicated that they feel different every day, so they did not like the idea of a static goal per day. Patients indicated that they preferred to be able to change the goal themselves because they do not see the health care professional often enough. However, changing goals should only be possible after a permissive message from the tool, to prevent downwards adjustments too easily.

#### Consequences for Goal Setting

Based on the results above, it was decided to set goals in a three-step process.

1. *Calibration*. During a two-week pre-measurement, the activity pattern of the users will be assessed, in order to set a realistic goal that is based on an objective measurement. In addition, the patient will receive questions (dialogue sessions) on the smartphone and website to identify which kind of activities the patient prefers and which barriers have to be overcome.
2. *Goal setting*. After the initial two weeks, the results of the pre-measurement will be evaluated by the practice nurse and discussed with the patient. Together, they will set an appropriate goal in minutes of moderate to vigorous physical activity per day. Appropriate means challenging, but within realistic margins, and personalized. Patients can adapt their goal themselves after a permissive message based on their activity results or by contacting the practice nurse.
3. *Activity planning*. Once at home, the patient will be invited by the tool to plan concrete daily activities to reach their goal. This will be facilitated by a dialogue session on the smartphone or on the website (at choice). This trigger to plan activities in detail (such as when, with whom and where you will be active) will narrow the gap between intention and behavior and make it more likely that the patient will reach the goal [47].

#### Feedback

There was consensus among respondents about how daily physical activity should be visualized on the smartphone. Activity performance should be set out against daily goals at any time. Performance data should be formatted as minutes of activity per day, rather than in calories (too complicated) or points (too abstract). Performance should be denoted in percentages of their goal, visualized in images, and color, but not using a childish animation. People did not state a preference to hear a sound when they reach their goal because it could interrupt them, could be noticed by others, and may become irritating. To monitor progress or decline, a historic overview of activity results over the last few months was deemed important. Some respondents indicated that they wanted to distinguish intensity rather than the type of activity (ie, sitting, biking, or swimming). Some respondents wanted to see a difference between moderately-intense and high-intensity activities, because this would stimulate them to do more high-intensity activities and they also wanted to receive more credits for those activities. Other respondents argued that they will feel the intensity themselves and that they will be happy to meet their goal anyway. This led to a lot of discussion during the focus groups and in the technical and research meetings. Another point of discussion was the choice between an absolute or relative threshold between the levels of intensity. Advocates of a relative threshold stated that everybody would experience high-intensity activities differently; for some chronically ill patients, a walking pace of 3 km per hour is exhausting, for others 5 km per hour is more appropriate*.* A relative threshold could be set per individual, based on a two week pre-measurement period. However, respondents with COPD also indicated that the difficulty of being active may differ from day to day.

Even for me, as an individual person, it is very hard to set a threshold. One day I am very fit, I exercise and nothing is wrong, the next day the ambulance is needed!

FG, COPD, male, 65 years

Everybody agreed that details about intensity need to be visible at a glance. Feedback messages from the server must be short, subtle, and positive in nature; without being paternalistic. Most respondents liked the idea that a health care professional could monitor their activity performance, because this would be an additional motivational factor. However, people thought they should also be able to make annotations to the activity data, in order to explain lower performance if one was sick (dyspnea), the weather was bad, etc.

Yeah, I think if a health care professional can watch your results that it has a psychological effect. You don’t want to disappoint the people who pay attention to you.

IR 1, DM2, female, 62 years

#### Consequences for Feedback

Based on the results above, it was decided that feedback will be given in 3 loops as already shown in Figure 2.

**Figure 3:**
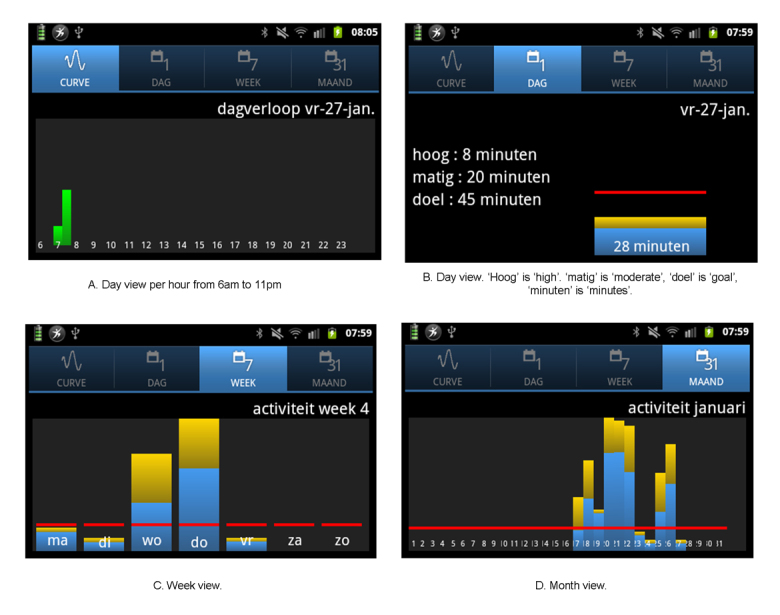
Activity menu on the smartphone app. The blue part of the bars indicates the moderately intense activity in minutes; the yellow part denotes the high-intensity activities. The red line indicates the daily goal.

1. In the first loop, data is directly visible on a widget on the smartphone. In a bar diagram, realized activity in minutes (≥3 Mets) is compared with the daily goal (see Figure 2). When the daily goal has been reached, a sun becomes visible as a subtle reward (see Figure 2). In addition, when opening the app, historic activity data can be viewed; in minutes per day, aggregated in days, weeks or months, as well as distinguishing between moderate (3-5.9 Mets) and intense (≥ 6 Mets) activities (see Figure 3).
2. In the second loop, periodic feedback messages will be sent after 3, 5, or 14 days. Various messages will be used, depending on the progress of performance, such as encouragement, positive trends, rewards, and suggestions to overcome barriers or to adjust personal goals.
3. In the third loop, users will receive feedback from the practice nurse. This will happen after 2 to 3 months and after 6 months, when the patient visits the practice nurse to evaluate the results and discuss barriers and facilitators. In between consultations, the practice nurse can monitor the activity results and is free to choose whether to react to this or not.

#### Data Sharing

Although most respondents perceived peer support as being important, only a few respondents were willing to share their activity results with relatives or peers. (eg, on a forum or social network, such as Facebook or Twitter.)

Sharing results on the internet is not motivating because the data and people are anonymous. Mutual support in real life is. And then you can decide to go hiking together.

IR2, male, DM2, 60 years

#### Consequences of Data Sharing

Activity data will only be shared with the practice nurse and not with peers. Sharing data through social media is not a priority. However, the involvement of relatives in the process of becoming more active will be encouraged in other ways.

#### Requirements of Health Care Professionals

Health care professionals admitted that they usually pay too little attention (approximately 10% of the consultation-time) to physical activity and that they welcome technological support to improve this. Most professionals viewed the tool as a mainly diagnostic instrument, since patients generally overestimate their level of physical activity. The activity pattern should be presented to them in time, intensity, steps, METS or calories, with aggregated information about the patients’ adherence and goal attainment. For their own convenience, they preferred to consult activity data within their own patient information system.

Regarding goal setting, professionals agreed on setting a goal together with the patient. Goals should be flexible, personal, and comorbidities should be taken into account. There was little to no enthusiasm, however, about the idea of giving feedback to the patients themselves in between consultations. An alternative idea of automated feedback messages was more appreciated. Professionals agreed with the patients that feedback messages should be positive (with a smiley face or flower), short and clear, but not pedantic. Performance should be visualized in numbers and graphs, including visible trends.

#### Design Principles Concerning the Requirements of Health Care Professionals

Based on the information above, it was decided that practice nurses will have their own web client (The “It’s LiFe!” Monitor) with two levels of information:

1. An overview window with aggregated information about the status of their patients’ goals.
2. A detail window presenting activity minutes per day and results from dialogue sessions.

With this information, the practice nurse can better prepare the patient consultation and can estimate appropriate patients’ goals more readily. The total physical activity counseling protocol will be published elsewhere. The integration with different patient information systems will not be realized during the “It’s Life!” project.

## Discussion

### Principal Results

In this study, the UCD process of a monitoring and feedback tool to enhance the self-management of physical activity for patients with COPD or type-2 diabetes is described. The research team gathered the user requirements and an engineering team translated these into technical solutions, to avoid data gathering with presuppositions. This tool is designed to be combined with a self-management support program for embedding in primary care. It provides a combination of behavior change techniques to increase knowledge, awareness (by self-monitoring) and self-efficacy. Personal goals are set and personalized feedback is provided based on the degree of goal attainment. The user-centered development process gave insight into the wishes and needs of the end-users, which will increase the likelihood of success. The main requirements for the tool derived from this process were:

- An activity sensor placed on the hip that measures activity accurately.
- Goals set in collaboration with the practice nurse after a pre-measurement period, in minutes activity per day. Personalized goals tailor the tool to individual needs.
- Feedback provided at different levels: immediate feedback, visible on the smartphone as a percentage of their daily activity goal, or presented as an image and in color; periodic feedback messages, always given with positive verbal; and aggregated feedback to the nurse practitioner, which should be used during the patient consultations.
- Activity data sharing with a care professional, not with peers on a forum or social media.
- An opportunity, for the patient to make annotations to their activity pattern.

Both end-user groups did not agree on all requirements. Patients want support from the practice nurse in between consultations, while care providers indicated that this is unmanageable due to time constraints. Therefore, automated feedback was incorporated to fulfill the need of patients of extra support. This makes it more suitable for daily practice in primary care. Most respondents were not open for sharing activity data on social media this may be influenced by the age of this group. The monitoring and feedback tool should be prepared for changes in this attitude.

### Limitations

A convenient sample of people with COPD or type-2 diabetes and health care professionals was used. Those who were interested in issues related to physical activity and/or technology may have been more likely to participate compared with others. Consequently, the study may have a self-selection bias. On the other hand, credibility [48] was increased by involving patient representatives in the research team in all decisions, and by the use of multiple data collection methods. Reliability was ensured by investigator triangulation, since the interviews were held by two different researchers and multiple researchers were involved in the analysis and the interpretation of data.

Other limitations included contextual restraints, such as budget, time, and the capabilities of technology in general and the engineering team. This led to some concessions, such as an activity sensor which is not waterproof, which mean there could be no registration of activity during swimming. Furthermore an activity sensor worn on the hip, is not able to register cycling. This may cause frustration among users that spend considerable time on these activities. It may also discourage users from developing these activities. The possibility to make annotations should compensate for this limitation.

### Comparison With Prior Work

Prior work has documented the effectiveness of pedometers to increase physical activity [16-18]. However, these were all short-term studies. It is unknown to what extent these changes are sustainable, since pedometers are still not routinely used in health care. An exceptional feature of our tool is the automated connection to the primary care professional via a secure website. Furthermore, the tool is embedded in a support program which is carefully aligned and simultaneously developed with the tool. Patients in this study indicated that the combination with coaching from the health care professional is a benefit, since health care professionals can serve as an extra motivator.

In this study, we developed a monitoring and feedback system in an iterative process inclusive of patients and health care professionals, to enhance the likelihood of success. While this study was conducted, a framework was published to improve the uptake and impact of eHealth technologies [49]. This framework, which is based on an extensive review, confirms the importance of end-users’ participation and an iterative development process. Furthermore, this framework emphasizes the importance of taking the conditions for implementation into account during the development process. In this study, this was achieved by involving health care professionals in the project and also by developing a self-management support program for the tool, which describes how the health care professional can support the patient.

The final design of the monitoring and feedback tool is in agreement with the proposed design strategies from Consolvo for technologies that support behavior change [50]. These strategies are based on the experiences from three persuasive technology interventions: Breakaway [51], Fish ‘n’ Steps [52] and Houston [23]. Consolvo’s 8 proposed strategies are: (1) Abstract & Reflective, (2) Unobtrusive, (3) Public, (4) Aesthetic, (5) Positive, (6) Controllable, (7) Trending/Historical, and (8) Comprehensive****.****

In addition, the developed tool, together with the self-management support program, is in line with Fogg’s theory of persuasive technology. According to Fogg, an intervention to change people’s behavior should focus on ability and motivation and provide a trigger to change [53]. Our intervention targets people who have the motivation to change their behavior but have not previously managed to do so. The self-monitoring tool makes people aware of their inactivity, which can lead to further motivation. The patient’s abilities are taken into account in the dialogue sessions, personal goals, and support from the practice nurse. In addition, our intervention provides a trigger to act by delivering feedback on physical activity on a timely basis and in an actionable format, namely related to tangible personal goals.

### Conclusions

In this paper, the development process of a monitoring and feedback tool is described as the preparation of an intervention to support the self-management of physical activity. It illustrates how a user-centered approach allows the consideration of valuable details to make the fit between the user, technology, and organization of care, which is important for the usability and acceptability of the tool. The leading principle of this intervention is to change behavior by self-monitoring, goal-setting, and feedback. The tool connects three technologies: an accelerometer, a smartphone app, and an Internet application. Feedback is given in three loops: direct feedback on daily activity compared with personal targets, periodic feedback on historical performance, and personal feedback by the practice nurse during consultations.

Having followed a user-centered design, we expect that the usability and acceptability of the tool has increased. This will be tested in a usability study in a lab environment and a pilot study in two general practices. The effect of the final tool embedded in primary care will be evaluated in a cluster randomized controlled trial.

## Multimedia Appendix 1

Results of three rounds of information gathering revealed from people with COPD or type 2 diabetes in stage 3, illustrating the dynamic specification process of requirements.

## Abbreviations

azM/Um: Univeristy Hospital Maastricht/Maastricht University
CAM: Ciro Activity Monitor
COPD: chronic obstructive pulmonary disease
DM2: type-2 diabetes
FG: focus group
IR1: interview round 1
IR2: interview round 2
NWO: The Netherlands Organization for Scientific Research
UCD: user-centered design
SSP: Self-management Support Program
ZonMw: The Netherlands Organization for Health Research and Development
