# Supplementary material for: The Development of a Mobile Monitoring and Feedback Tool to Stimulate Physical Activity of People With a Chronic Disease in Primary Care: A User-Centered Design
Source: JMIR Mhealth Uhealth. 2013 Jul 2;1(2):e8. doi: 10.2196/mhealth.2526 (PMC4114510; doi:10.2196/mhealth.2526)
Supplement: Supplementary file 1 [file mhealth_v1i1e8_app1.pdf]

Attachment 1. Results of three rounds of information gathering revealed from people with COPD or type 2 diabetes in stage 3, illustrating the dynamic specification process of requirements.

Interview topics	Results 1st interview round	Results 2nd interview round	Results focus groups
**Tool architecture**			
**Place activity sensor**	On Shoe*<br>On Hip with strap or clip*<br>Around leg*<br>In watch/bracelet*<br>Necklace<br>In glasses<br>In shoe soles<br>Implant<br>On bra-strap<br>In inhaler [47]<br>On insulin pump (DM2)<br><br>* these options were proposed in the second interview round	On hip with strap or clip<br>In watch	Hip
**Requirements**	Consistent measurement	Lightweight	Consistent

**activity sensor**	Not hinder movements<br>Unobtrusive<br>Design not important<br>Hygienic<br>You should not be able to cheat<br>Waterproof	Shockproof<br>Not hinder movements<br>Unobtrusive for yourself and others<br>Design not important<br>Neutral color (possibly leather or fabric around it)<br>Requirements for activity sensor on hip:<br>Large clip and no strap<br>Not wider than belt	measurement<br>Shockproof<br>Not hinder movements<br>Unobtrusive for yourself and others<br>Design not important<br>Large clip<br>Not wider than belt<br>Hygienic<br>No interference with insulin pump<br>Indication if battery is low<br>Battery life of a week<br>Case for device to attach to belt<br>Waterproof
**Goal setting**			
What	Day goal<br>Week goal<br>Personally relevant goal	Day goal<br>Personally relevant goal	Day goal

Who	In collaboration with care professional<br>Independent	In collaboration with care professional	In collaboration with care professional
Change goal	In collaboration with care professional<br>Independent	independent after message tool	In collaboration with care professional<br>Independent after message tool

Feedback

1. Amount of activity

What	Minutes<br>Steps<br>Heart rate<br>Kilometers<br>Relation of activity with blood glucose levels and apnea<br>Activity history to see pattern and decline in activity	Minutes total<br>Difference in intensity<br>Heart rate<br>Activity history on the computer	Minutes<br>Activity history
Where	Activity sensor<br>Mobile phone<br>Computer<br>By mail	Mobile phone<br>Computer	Mobile phone
Feedback		Possibility to annotate	

requirements		the activity data	
**2. Amount of activity compared to goal**			
What	Achieving goal yes/no<br>Percentage of goal achieved	Percentage of goal achieved	Percentage of goal achieved<br>Achieving goal yes/no<br>Feedback messages
Where	Activity sensor<br>Mobile phone<br>Computer<br>By mail	Mobile phone<br>Computer	Mobile phone
How	Graph<br>Picture<br>Text<br>Numeral<br>Percentage<br>Scale (0-100)<br>Sound<br>Color<br>Cheering<br>Vibration<br>Lights	Graph<br>Numeral<br>Percentage<br>Color<br>Vibration	Text<br>Percentage<br>Color<br>Image
**3. Response of a health professional based on the activity results**			

Who	Practice nurse<br>General practitioner<br>Physiotherapist<br>Diabetes nurse<br>Internist<br>Pulmonary nurse	Practice nurse<br>General practitioner	Practice nurse
How	By phone<br>In consultation	By phone<br>In consultation	By phone<br>In consultation
Effect	Extra motivator<br>One person does not like it	Extra motivator<br>One person: Only if I bring the results myself	Extra motivator
**Data sharing**			
Share activity results with peers	No need to do this automatically, only in real life	No need to do this automatically, only in real life	No need to do this automatically, only in real life
Share activity results with relatives	No need to do this automatically (Facebook, Twitter etc.) only in real life	No need to do this automatically (Facebook, Twitter etc.) only in real life	No need to do this automatically (Facebook, Twitter etc.) only in real life
